# Ligand-Induced Conformational Dynamics of A Tyramine Receptor from *Sitophilus oryzae*

**DOI:** 10.1038/s41598-019-52478-x

**Published:** 2019-11-07

**Authors:** Mac Kevin E. Braza, Jerrica Dominique N. Gazmen, Eizadora T. Yu, Ricky B. Nellas

**Affiliations:** Institute of Chemistry, College of Science, University of the Philippines Diliman, Quezon City, 1101 Philippines

**Keywords:** Computational biophysics, Protein structure predictions

## Abstract

Tyramine receptor (TyrR) is a biogenic amine G protein-coupled receptor (GPCR) associated with many important physiological functions in insect locomotion, reproduction, and pheromone response. Binding of specific ligands to the TyrR triggers conformational changes, relays the signal to G proteins, and initiates an appropriate cellular response. Here, we monitor the binding effect of agonist compounds, tyramine and amitraz, to a *Sitophilus oryzae* tyramine receptor (SoTyrR) homology model and their elicited conformational changes. All-atom molecular dynamics (MD) simulations of SoTyrR-ligand complexes have shown varying dynamic behavior, especially at the intracellular loop 3 (IL3) region. Moreover, in contrast to SoTyrR-tyramine, SoTyrR-amitraz and non-liganded SoTyrR shows greater flexibility at IL3 residues and were found to be coupled to the most dominant motion in the receptor. Our results suggest that the conformational changes induced by amitraz are different from the natural ligand tyramine, albeit being both agonists of SoTyrR. This is the first attempt to understand the biophysical implication of amitraz and tyramine binding to the intracellular domains of TyrR. Our data may provide insights into the early effects of ligand binding to the activation process of SoTyrR.

## Introduction

Octopamine (OA) and tyramine (TA) are tyrosine-derived biogenic amines that are vital to many physiological processes in insects. They are analogous to the effect of adrenaline and noradrenaline in vertebrates as neurotransmitters in the human nervous system^1,2^. They are found in high concentrations in the nervous systems of invertebrates acting as neurotransmitters, neuromodulators, and neurohormones in insects^3,4^. As neurotransmitters, they are known to regulate endocrine gland activity in fireflies (*Photuris versicolor*) and California sea hare (*Aplysia californica*)^5,6^. As neuromodulators, OA is involved in the regulation and desensitization of sense organs, and regulation of rhythmic and complex behaviors such as memory and learning in fruit fly (*Drosophila*)^7^. As neurohormones, OA stimulates metabolism, inducing mobilization of lipids and carbohydrates in insects^2^. OA and TA elicit physiological effects through binding to specific aminergic receptors. These seven-transmembrane protein receptors belonging to the class A G protein-coupled receptor (GPCR) family mediate signal transduction via an intracellular heterotrimeric G protein. These octopamine and tyramine receptors (OctR and TyrR, respectively) are attractive targets in the development of bioactives against insects since their occurrence is restricted to invertebrates only^8,9^. Despite the existence of pesticides that target said receptors in insects, the lack of a molecular understanding of receptor-pesticide interactions limits the tailored design of pesticides for specific aminergic receptors^10,11^.

In order to elucidate molecular mechanisms of ligand specificity and activation, it is essential to investigate signature receptor residues that interact with such ligands. The binding site of octopamine and tyramine receptors is said to comprise of the following signature amino acids: (a) a negatively charged Asp in TM3 that interacts with the protonated amine group of the ligand^12–14^; (b) a closely spaced Ser in TM5 that interacts with the phenolic hydroxyl group of the ligand^14–16^; and (c) an aromatic cluster in TM6 that exhibits *π* − *π* or hydrophobic interactions with the aromatic group of the ligand^17,18^. In addition, specific for OctR, a Tyr residue in TM6 interacts with the *β*-hydroxyl group of OA. The identity of these contact sites seems to indicate that the role of the biogenic amine is to trigger communication between TM3, TM5, and TM6^19,20^. These conformational dynamics elicit an overall conformational change in OctR and subsequently induce downstream G protein-dependent signaling. Tyr in TM6 is suspected to mediate a conformational change between semi-active and fully active states in OctR. This molecular switch exhibits dynamic H-bonding with the *β*-hydroxyl group of OA^21^. While the said switch is conserved at an equivalent position in TyrR, it is interesting to note that TA does not have a *β*-hydroxyl group. Thus, activation in TyrR might proceed via a mechanism different from that proposed for OctR.

Aside from the knowledge of ligand binding sites, structural information is needed to elucidate a detailed molecular mechanism of TyrR activation. However, there are no solved crystal structures for OctR and TyrR to this date because the majority of their surfaces are buried inside a membrane. In this regard, alternative methods such as homology modeling and molecular docking studies are undertaken for structural characterization of membrane proteins. Molecular dynamics (MD) studies, on the other hand, have become a powerful tool in elucidating membrane protein behavior in their cellular environment^22,23^. Several studies have used MD simulations in distinguishing the effects of different ligands to the activation of other related GPCRs, e.g. serotonin and adenosine receptor^24–27^. These parallel studies have proven the importance of computational studies in explaining the ligand-dependent mechanism of various GPCRs.

Experimental studies have been conducted on TyrR of other insects including silkworm (*Bombyx mori*), honeybee (*Apis mellifera*), cockroach (*Periplaneta americana*), cattle tick (*Rhipicephalus microplus*), and fruit fly (*Drosophila melanogaster*)^13,14,28–31^. These studies have mainly focused on physiological function, ligand binding, and binding site identification. To the best of our knowledge, there have been no attempts to explain the molecular mechanism of ligand activation of TyrR. In this study, we probe to understand the early structural effects of ligand modulators to rice weevil (*Sitophilus oryzae*) tyramine receptor (SoTyrR). Through MD simulations of SoTyrR homology model, we attempt to describe ligand-induced conformational dynamics of SoTyrR. We surmised that the importance of correlated motions within the receptor and the interplay of the amino acid residues in TMs and IL3 domain influences the activation process of SoTyrR in agreement with the general mechanism of GPCR activation.

## Results

### Homology modeling

**Figure 1:**
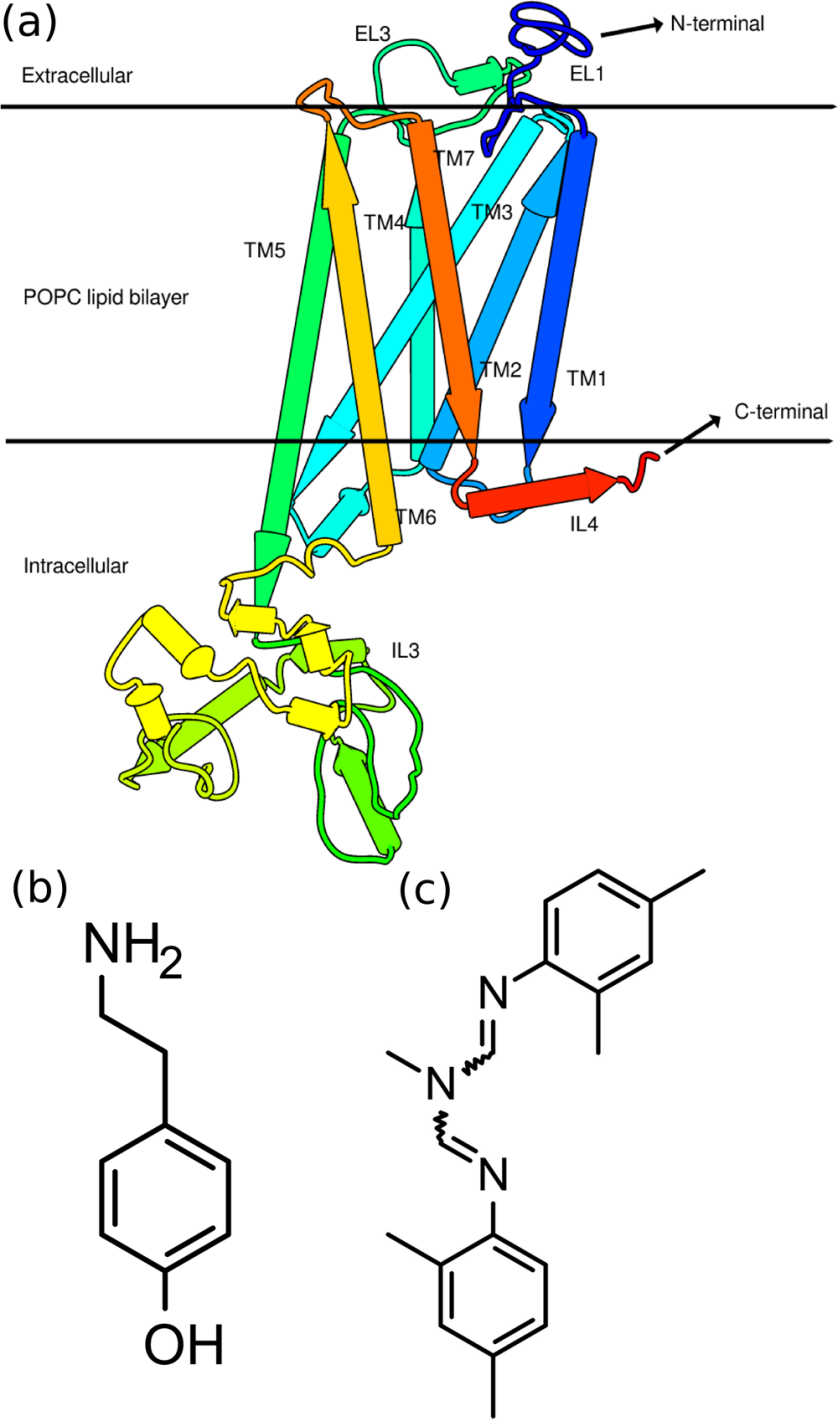
(**a**) Homology model of SoTyrR from I-TASSER. Chemical structures of tyramine receptor ligands, (**b**) tyramine (natural agonist) and (**c**) amitraz (agonist).

In the absence of crystal structure, we built the 3D homology model of *Sitophilus oryzae* tyramine receptor (SoTyrR) using the online server I-TASSER (Fig. 1a)^32,33^. Here, various GPCR crystal structures with 23 to 32% sequence identity were used as template. As previously observed for this group of proteins, a higher structural and sequence similarity is expected in the transmembrane (TM) region than in the extracellular and intracellular loops^34,35^. As obtained from I-TASSER, the model of SoTyrR has a C-score of −0.87 and an estimated TM-score (Template modeling-score) of 0.60 ± 0.14. C-score value represents the prediction accuracy of the model based on the quality of threading alignments and the convergence of the simulated structures^36^. A C-score value of −1.5 or higher depicts correct folding of the protein^36^. On the other hand, TM score is defined as the quality of matching among protein of native and predicted structures. A TM-score value >0.5 represents similar fold^32,33,36^. Although, the query and target sequence have low % sequence similarity, the obtained SoTyrR 3D structure adequately represents the correct topology of protein especially at the transmembrane region where the natural ligand binds^37^.

Other homology modeling servers, GPCR-I-TASSER and SWISS-MODEL, were also explored for modeling the SoTyrR 3D structure. The I-TASSER result predicts better stereochemical property than the GPCR I-TASSER model and provides complete modeled residues as compared to the 57 missing residues from the SWISS-MODEL result. Based on the stereochemical quality (Ramachandran plot) prediction and minimal missing residues modeled, the I-TASSER result provides a reasonable starting model for SoTyrR MD simulations.

### Molecular affinity of ligands to SoTyrR

**Figure 2:**
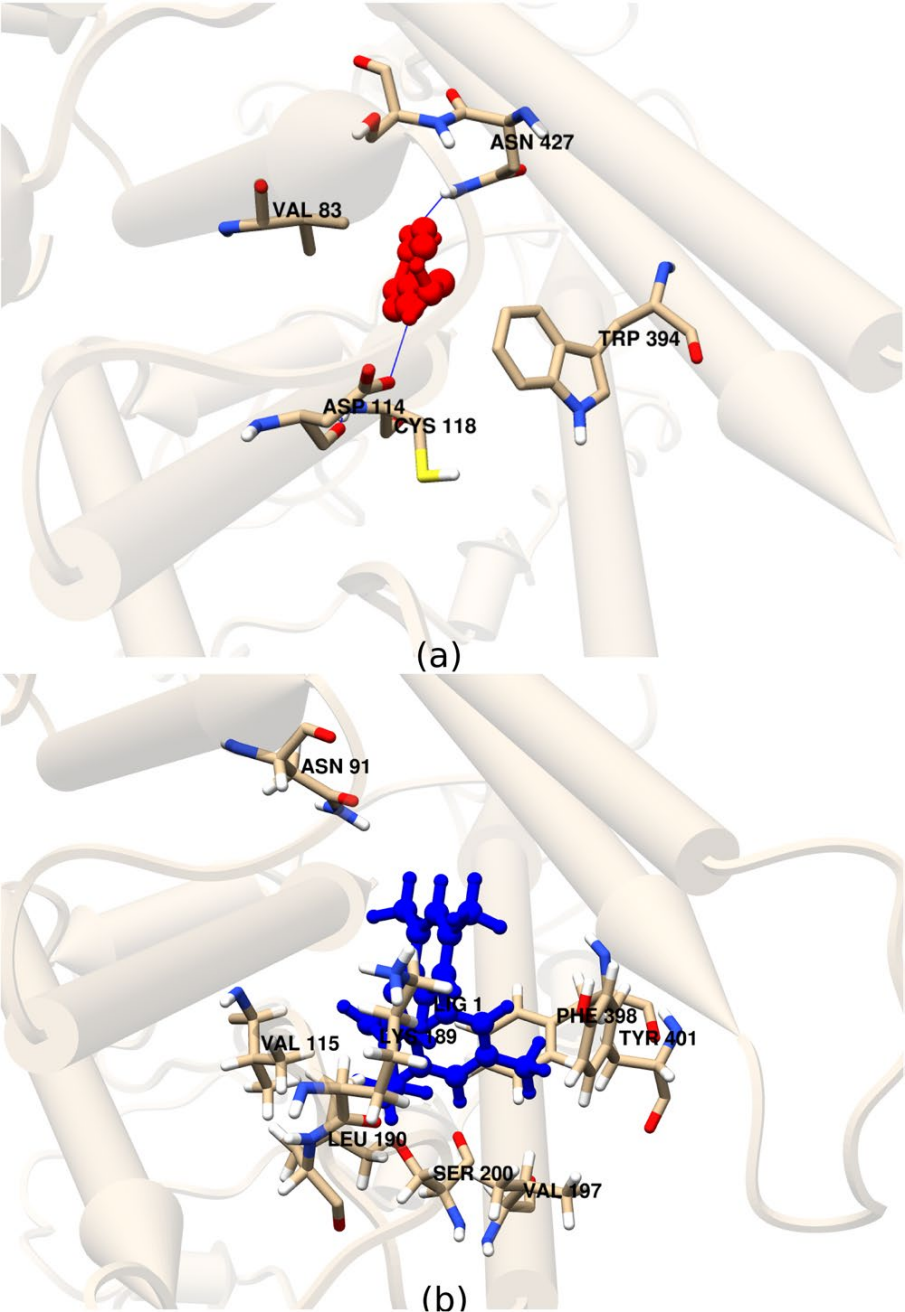
Close interacting residues of SoTyrR with ligands (ball-and-stick representation), (**a**) tyramine (red) and (**b**) amitraz (blue).

Tyramine and amitraz (Fig. 1b,c) are known activators of octopaminergic receptors. Ensemble docking of ligands tyramine and amitraz to SoTyrR was done using AutoDock VINA^38^. An ensemble of receptor conformations was obtained from the 100-ns simulation in aqueous 1-palmitoyl-2-oleoyl-sn-glycero-3-phosphocholine (POPC) lipid bilayer and the binding energy was reported as an average of the ensemble docking results. The close-contacting residues of SoTyrR in each protein-ligand complex are summarized in Table 1 and the binding pockets for each compound are presented in Fig. 2. It can be seen that the binding site for tyramine resides in the core of transmembrane bundles comprising of residues in TM2, 3, 6, and 7. Tyramine was observed to form an H-bonds to SoTyrR Asp114 (in TM3) and Asn427 (in TM7). A previous mutational study suggests that a conserved Asp in TM3 is important in the ligand specificity of tyramine receptors by H-bonding with the amine group of the ligand^14^. On the other hand, amitraz complexed to SoTyrR has a different binding region, albeit close to the orthosteric binding site. As shown in Fig. 2b, amitraz is in contact with residues Asn91, Val115, Lys189, Leu190, Val197, Ser200, Phe398, and Tyr401 at TM2, 3, and 6. Despite the known effects of amitraz binding to SoTyrR, studies on the actual binding site of Tyr is lacking^39,40^. Based on the binding energy, the *K*_*i*_ value was calculated using this equation: ${K}_{i}=\exp \,[\Delta G{R}^{-1}{T}^{-1}]$ where Δ*G* is the binding energy in cal · mol^−1^, R is the gas constant equal to 1.9187 cal · mol^−1^ · K^−1^, and T is the temperature equal to 300 K. Tyramine has *K*_*i*_ value of 85.4 *μ*M and amitraz has 1.59 *μ*M.

**Table 1:** Close-interacting residues of SoTyrR from molecular docking of agonist ligands. Figure 2 shows the corresponding visual representations.

System	Binding site residues	Binding energies, kcal/mol*
SoTyrR-tyramine	Asp114 (H-bonding), Val83, Cys118, Trp394, Asn427, and Ser428	−5.55 ± 0.32
SoTyrR-amitraz	Asn91, Val115, Lys189, Leu190, Val197, Ser200, Phe398, and Tyr401	−7.91 ± 0.28

*Binding energies reported as Ave ± SD of ensemble docking to 100 clustered structures of SoTyrR from 100-ns MD simulations.

### Conformational analyses of SoTyrR-ligand systems

#### RMSD analysis

**Figure 3:**
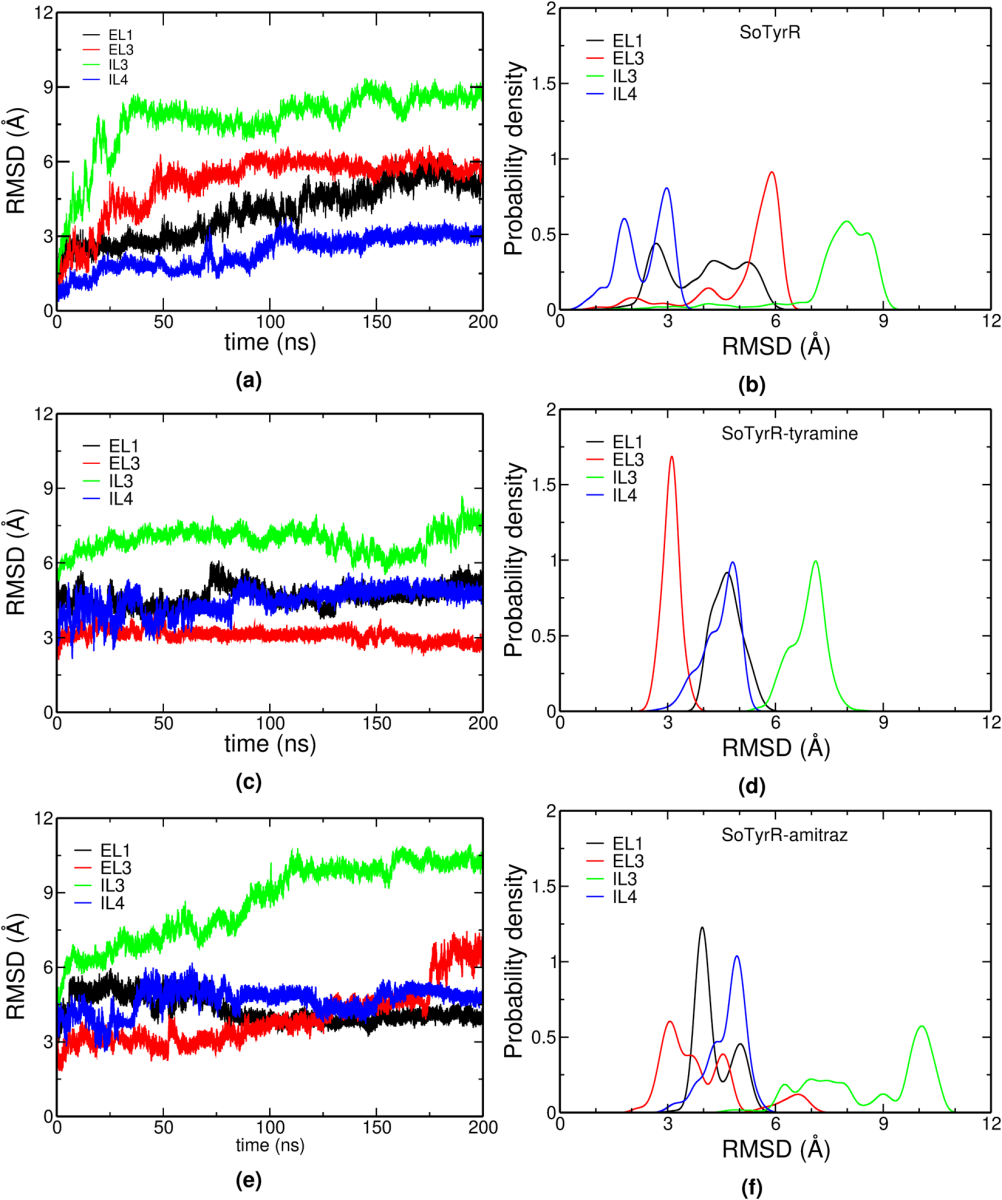
Domain-based protein backbone RMSD and the corresponding RMSD distributions for (**a**,**b**) SoTyrR, (**c**,**d**) SoTyrR-tyramine, and (**e**,**f**) SoTyrR-amitraz, respectively. The first frame of SoTyrR (apo) was used as the reference point for calculations. (Notations: EL1 = Extracellular loop 1, EL3 = Extracellular loop 3, IL3 = Intracellular loop 3, and IL4 = Intracellular loop 4).

Molecular dynamics simulations of protein-ligand complexes were done in NAMD 2.11^41^ for 200 ns. Root-mean-square deviation (RMSD) measures structural changes with respect to a reference conformation (first frame in the production run of SoTyrR). The average RMSD at each protein domain is summarized in Table 2. Unlike in the loop regions in the intracellular and extracellular sides of SoTyrR, the transmembrane (TM) helices have a maximum of 3 Å RMSD. The IL3 region has the greatest average RMSD recorded in all of the SoTyrR-ligand systems. Also, the EL1, EL3, and IL4 have shown significant changes in the three SoTyrR complexes. After plotting the probability densities of their RMSD, as depicted in Fig. 3, the changes of most populated RMSD become more evident. For instance, the EL3 region (RMSD = 3Å) in SoTyrR-tyramine is the most populated RMSD value, but for SoTyrR-amitraz and SoTyrR, the values become less populated and widespread.

**Table 2:** Average RMSD (Å) per domain of SoTyrR-ligand complexes.

Domain	SoTyrR	SoTyrR-tyramine	SoTyrR-amitraz
EL1	3.94 ± 1.10	4.66 ± 0.40	4.30 ± 0.52
EL2	1.03 ± 0.13	1.43 ± 0.16	1.67 ± 0.17
EL3	5.17 ± 1.20	3.10 ± 0.23	3.95 ± 1.14
EL4	1.70 ± 0.36	3.71 ± 0.73	3.74 ± 0.44
IL1	1.29 ± 0.58	1.90 ± 0.18	1.85 ± 0.26
IL2	0.95 ± 0.26	1.76 ± 0.19	2.03 ± 0.47
IL3	7.65 ± 1.33	6.90 ± 0.48	8.64 ± 1.55
IL4	2.33 ± 0.68	4.46 ± 0.51	4.66 ± 0.52
TM1	1.80 ± 0.32	2.03 ± 0.23	2.05 ± 0.23
TM2	1.13 ± 0.24	1.89 ± 0.16	1.64 ± 0.29
TM3	0.63 ± 0.13	0.94 ± 0.14	0.70 ± 0.10
TM4	0.70 ± 0.12	2.03 ± 0.27	1.51 ± 0.27
TM5	1.04 ± 0.26	1.12 ± 0.08	1.37 ± 0.12
TM6	0.96 ± 0.27	1.07 ± 0.35	1.57 ± 0.47
TM7	1.56 ± 0.28	2.48 ± 0.25	1.57 ± 0.17

#### B-factor values

**Figure 4:**
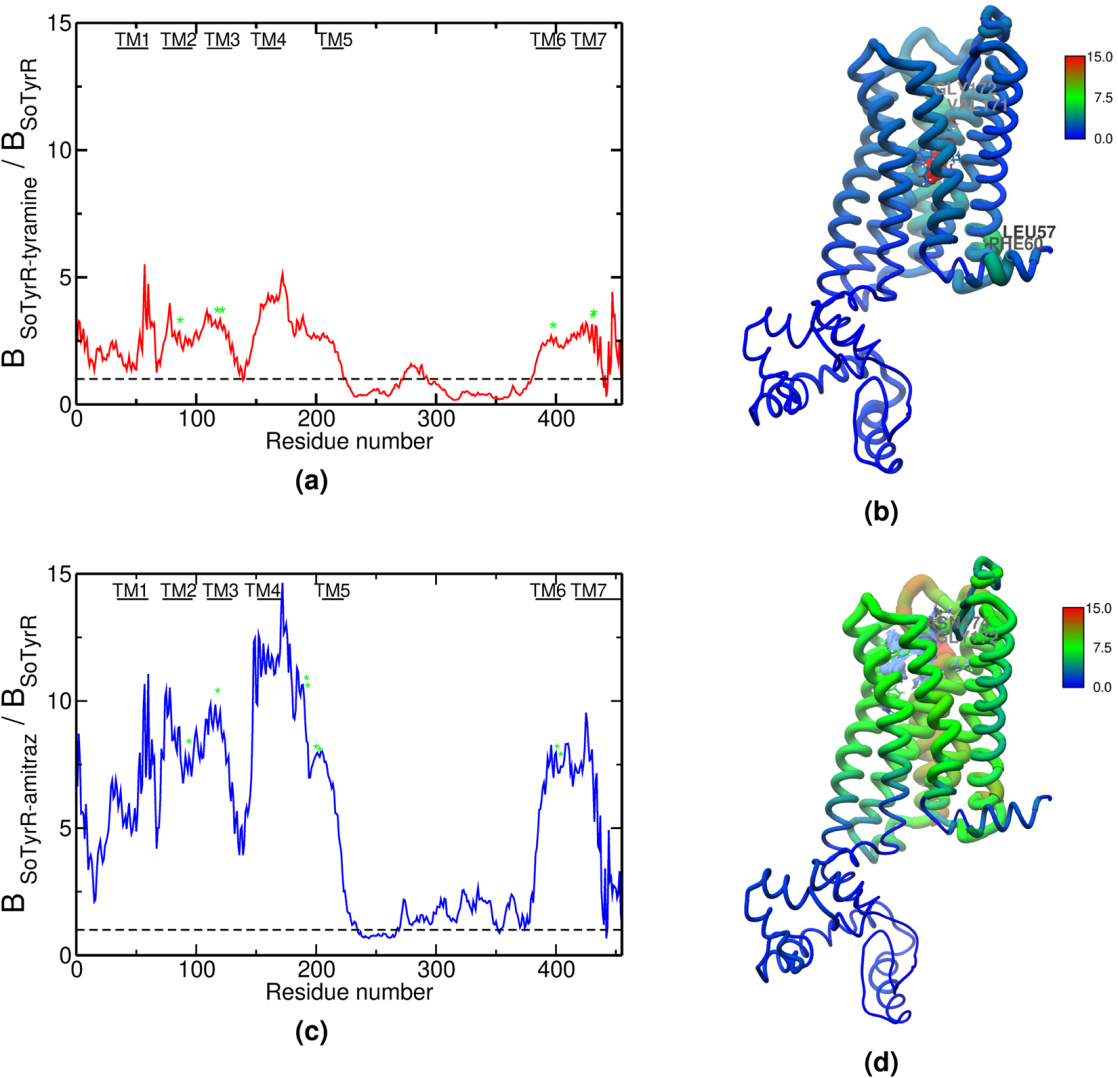
Protein backbone B-factor ratio and 3D B-factor projection for (**a**,**b**) SoTyrR-tyramine:SoTyrR and (**c**,**d**) SoTyrR-amitraz:SoTyrR, respectively. Close-interacting residues are labeled with green asterisks.

Local motions of the SoTyrR were measured by Debye-Weller factor or B-factor. Here, mean-square fluctuation B-factor was calculated using this equation: $B=[8{\pi }^{2}(\Delta {r}^{2})]/3$ where *r* is the coordinate of the atom obtained from the trajectories of SoTyrR protein backbone. The results are shown in Fig. 4. In both SoTyrR-tyramine and SoTyrR-amitraz, the IL3 and IL4 regions are found to be more unperturbed than in the apo state. Moreover, the TM regions become more flexible in SoTyrR-amitraz than in SoTyrR-tyramine as evident in the higher B-factor ratios above 1.00. Several residues in the ligand binding sites including Asp114 in SoTyrR-tyramine have exhibited high B-factor values in spite of their interactions with the ligand. Similar observations were found for SoTyrR-amitraz at residues Asn91, Lys189, Leu190, Val197, and Tyr401.

#### Principal component analyses (PCA)

**Figure 5:**
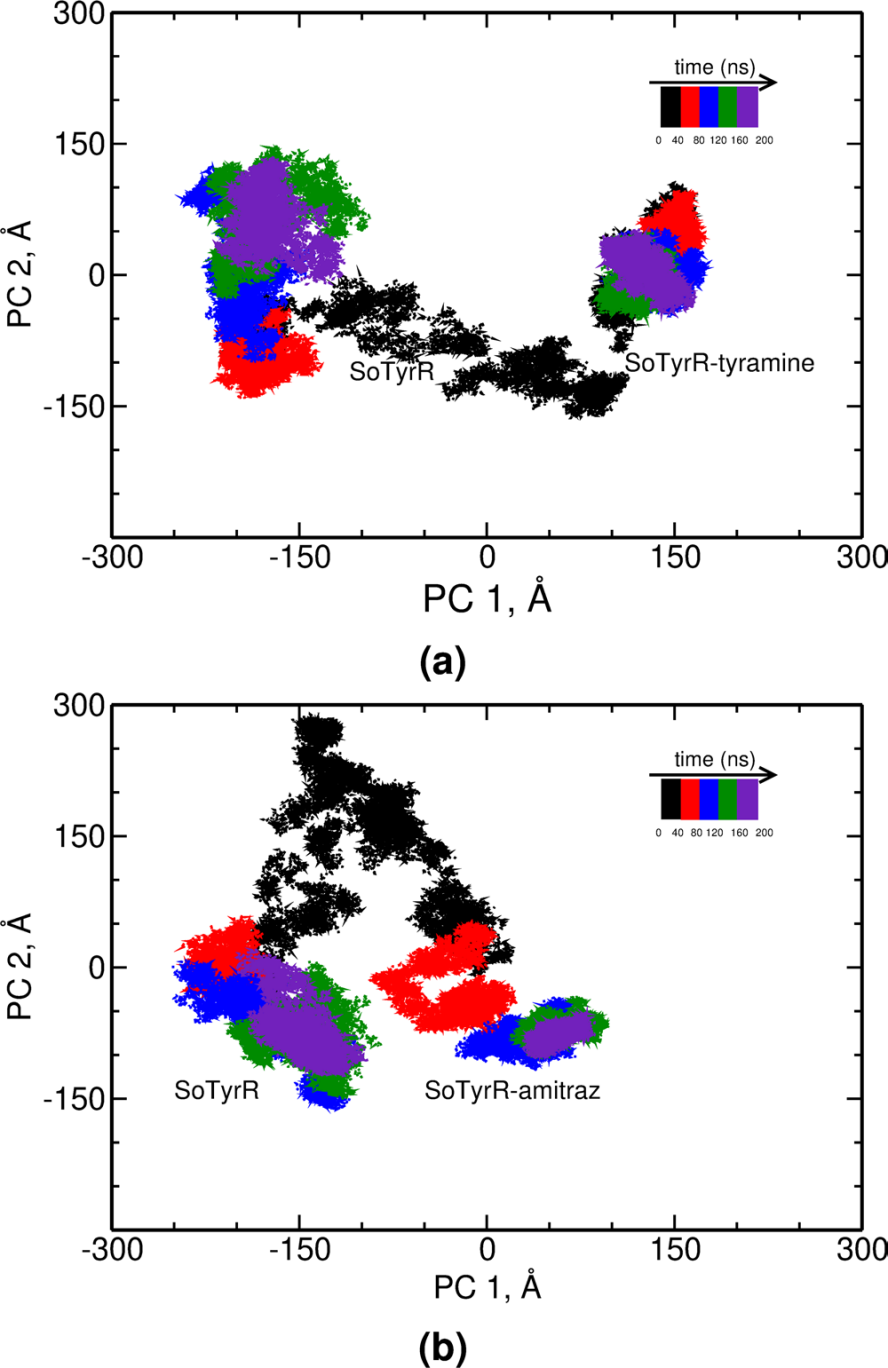
Principal component analyses for (**a**) SoTyrR-tyramine:SoTyrR and (**b**) SoTyrR-amitraz:SoTyrR. The subspace sampled at every 40 ns is colored accordingly.

Principal component analyses were used to identify the collective motion of the protein from a large number of conformations visited throughout the course of MD simulations^42,43^. The two most dominant PCs of SoTyrR-ligand complexes are projected in Fig. 5 with respect to SoTyrR PCs (i.e. mixed PCAs). To get the most populated PCs of the motions sampled, the free energy of the binned population was calculated using ${G}_{i}=-\,{k}_{B}T\,\mathrm{ln}\,({N}_{i}/{N}_{{\max }})$ where *k*_*B*_ is the Boltzmann’s constant, *T* is temperature equals to 300 K, *N*_*i*_ is the population of bin *i* and *N*_*max*_ is the population of the most populated bin as reported in Supplementary Information Fig. 2. Lower free energy values signify a more populated PC.

After employing mixed PCA, in SoTyrR-tyramine complex, the PC 1 and 2 corresponds to the 33 and 24%, respectively, of the overall motion of the protein backbone as reported in Table 3. By comparing the values of the top two PCs with respect to SoTyrR, SoTyrR-amitraz exhibits higher percentages with 39 and 24% PC 1 and 2, respectively.

**Table 3:** The percent contribution of principal components, PC 1 to PC 5, to the overall motion of SoTyrR.

System	PC 1	PC 2	PC 3	PC 4	PC 5
SoTyrR-tyramine	33.44	24.30	21.63	14.70	5.93
SoTyrR-amitraz	38.87	23.62	17.20	14.02	6.28

Based on the time-evolution fluctuations, it can be seen that the convergence at SoTyrR-tyramine starts as early as 20 ns while for SoTyrR-amitraz, convergence starts at around 80 ns. In SoTyrR-tyramine, the PC 1 values range from 100.0 to 150 Å. For SoTyrR-amitraz, majority of PC 1 lies on −150 to 100 and has wide-range PC 2 values of −150 to 300 Å. Based on the free-energy plot of PCs population (SI Fig. 2), the PC 1 values that are most populated in SoTyrR-tyramine and SoTyrR-amitraz were recorded at around 150.0 and 100.0 Å, respectively.

**Figure 6:**
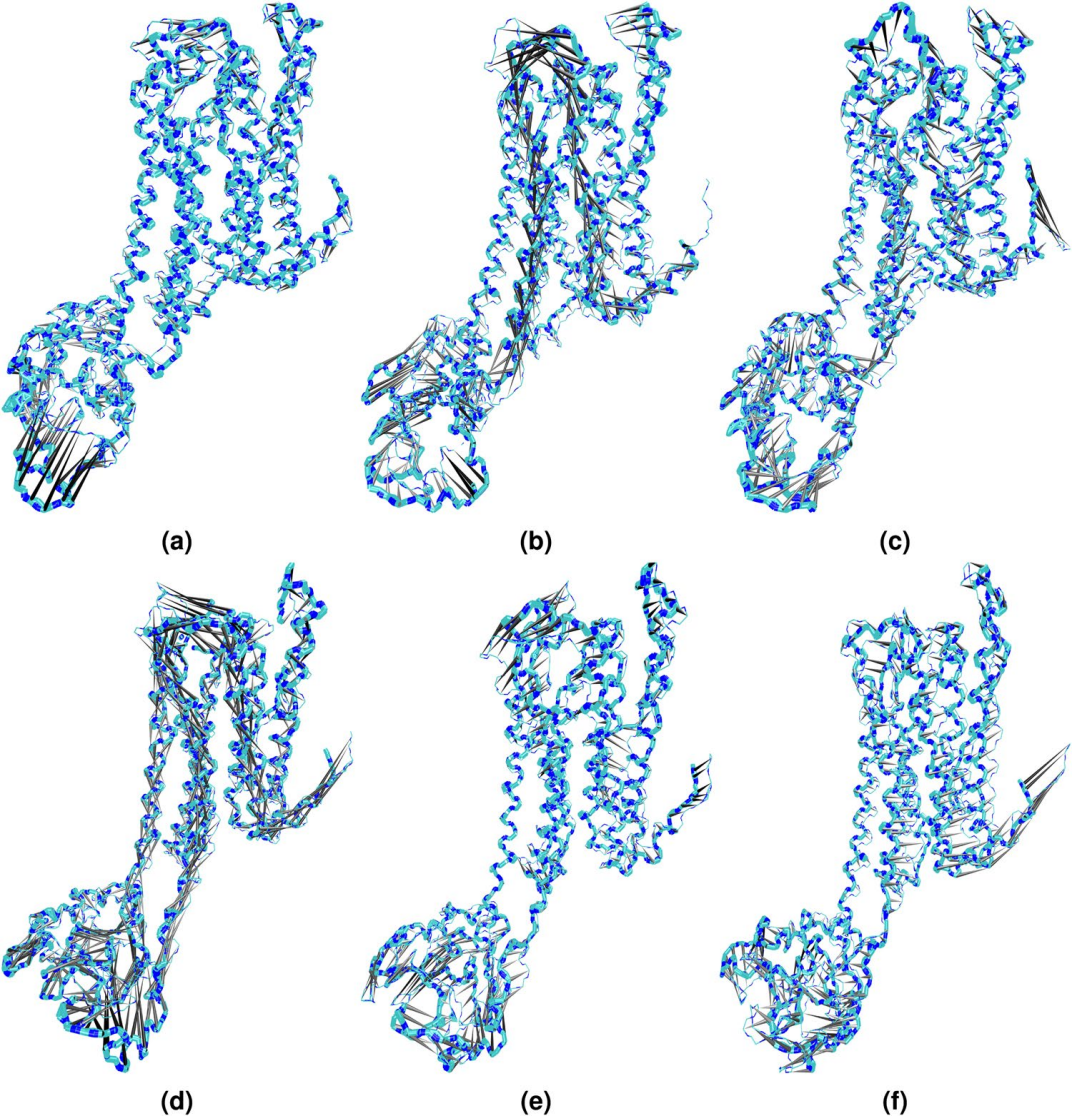
3D projections of mixed principal components using porcupine plot of SoTyrR-ligand complexes. (**a**) PC 1; (**b**) PC 2; and (**c**) PC 3 of SoTyrR-tyramine; (**d**) PC 1; (**e**) PC 2; and (**f**) PC 3 of SoTyrR-amitraz. Largest displacement is colored as black.

The corresponding motions of PC 1, 2, and 3 were also projected in SoTyrR (Fig. 6). The porcupine plot shows the conformational displacement in C*α's* of SoTyrR-ligand complexes at different PCs. Based on the 3D projection of SoTyrR, the largest contributor in the displacement was in IL3 region. It is further emphasized in PC 2 where concerted movement of TM5 and EL3 is also observed, especially in SoTyrR-tyramine.

#### Correlation matrix and network analyses

Here, we also identify the communication in the protein receptor by determining correlated dynamic motions. The correlation matrix analyses can be found in SI Fig. 1. The plot identifies the residues that are positively- and/or negatively-correlated at different SoTyrR-ligand complexes. Regions in red correspond to strong positive correlated-residues and regions in purple color have negative correlation.

For instance, in the SoTyrR and SoTyrR-tyramine complex, it can be seen that residues found in the IL3 (residues 224–382) show anticorrelation with those residues found in the EL2 (residues 98–108), TM3 (residues 109–130) and EL3 residues (residues 172–204). Residues 300–380 become positively correlated with residues 210–220 after ligand-binding (absent in SoTyrR). A similar trend of conformational correlations was observed with SoTyrR-amitraz but with more pronounced changes at the same amino acid residues as in SoTyrR-tyramine. Dynamic correlations in the protein domains have revealed that the strongest couplings are found in the intracellular region.

**Figure 7:**
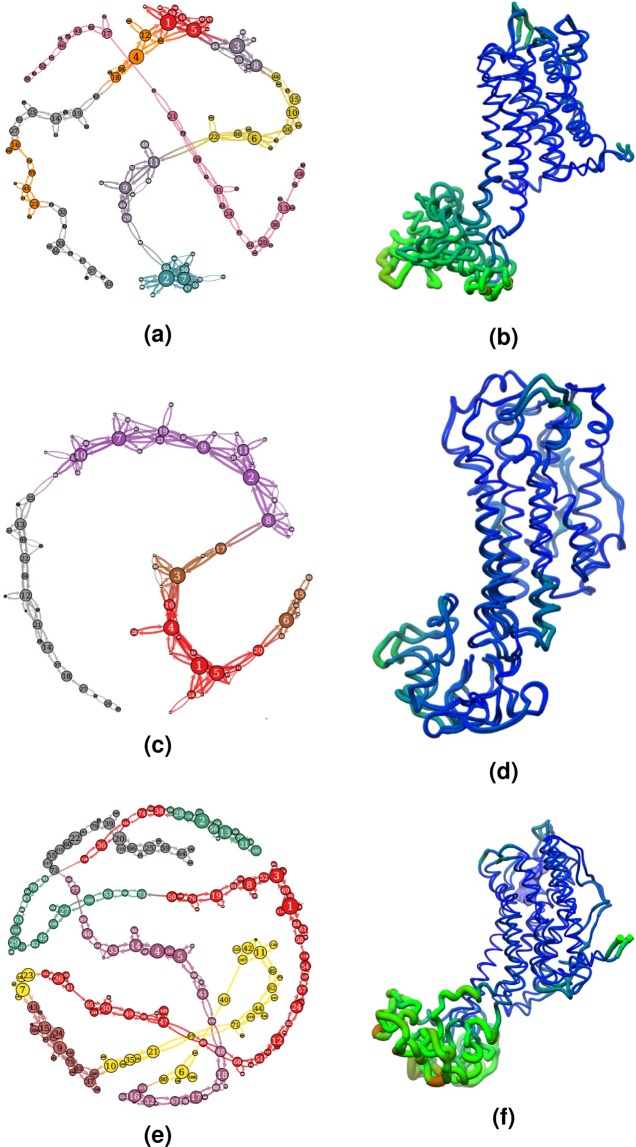
Network representation of conformational transitions of SoTyrR within 1–100 ns simulations. (Left) Network profile of clustered structures of (**a**) SoTyrR, (**c**)SoTyrR-tyramine, and (**e**) SoTyrR-amitraz systems. (Right) Superimposition of structures from nodes 1 and 2 in (**b**) SoTyrR, (**d**) SoTyrR-tyramine, and (**f**) SoTyrR-amitraz. The thickness of worm representation depicts the RMSD value between the two structures.

**Figure 8:**
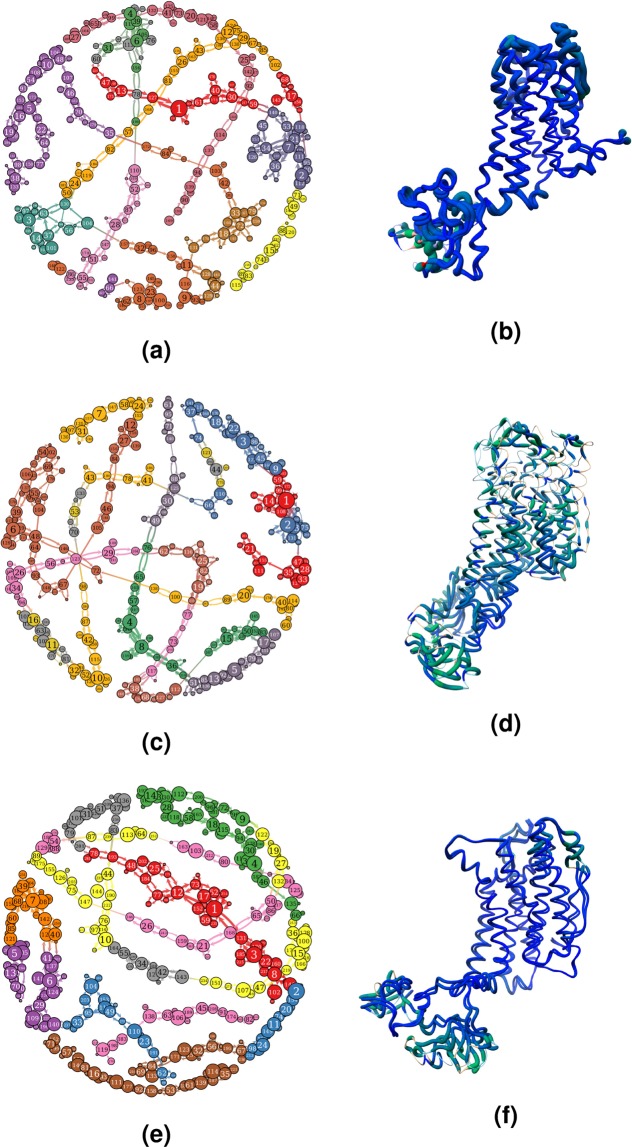
Network representation of conformational transitions of SoTyrR within 101–200 ns simulations. (Left) Network profile of clustered structures of (**a**) SoTyrR, (**c**)SoTyrR-tyramine, and (**e**) SoTyrR-amitraz systems. (Right) Superimposition of structures from nodes 1 and 2 in (**b**) SoTyrR, (**d**) SoTyrR-tyramine, and (**f**) SoTyrR-amitraz. The thickness of worm representation depicts the RMSD value between the two structures.

The network representations of SoTyrR conformational transitions are shown in Figs 7 and 8. Structurally similar conformations of the protein were clustered together forming the nodes of the network. Observed transitions in the MD simulations represent the edges connecting the nodes. Moreover, the most visited conformation of SoTyrR in each liganded system contains the largest node. Based on the number of partition states distributed (depicted as color groups in the nodes), the SoTyrR-tyramine complex has only four most visited conformations. While, SoTyrR and SoTyrR-amitraz have seven different partition states visited. It has been confirmed that the largest displacement were found in the EL and IL regions, while there are very minimal changes in the TM sections. The differences in the structures are shown and compared with three most visited conformations of SoTyrR at each different system by using RMSD metrics. Findings in this study showed that the most notable difference among the top clustered structures are found in IL3 region as shown in both Figs 7 and 8. The RMSD between first and second structure’s IL3 in SoTyrR and SoTyrR-amitraz have values greater than 3 Å.

## Discussion

The G protein-coupled receptors (GPCRs) participate in many cell signaling processes and physiological functions in the cell. Within the GPCR protein family, each receptor type undergoes activation pathway differently despite of their ability to recognize same set of intracellular G proteins^44,45^. This leads to the question on how the activation profile of two agonists changes the transformation of inactive to active state of the protein after ligand binding. In this study, the tyramine receptor from rice weevil (*Sitophilus oryzae*), SoTyrR, was subjected to biophysical probing to understand the dynamic behavior of insect receptor with or without the presence of an agonist ligand. Although several research groups have explored the binding site, dynamics behavior, and post-binding effect of octopamine receptors from different insects, this is the first attempt to correlate intradomain dynamics of SoTyrR to understand probable early effects of tyramine and amitraz binding to tyramine receptor’s activation^46–49^.

Several neurotoxic compounds acting against insect receptors were discovered several years ago. Many of these compounds against pests have targeted neuroactive insect receptors such as acetylcholinesterase, nicotinic receptors, and octopaminergic receptors (e.g. tyramine receptor)^50^. It has been well established that amitraz has insect repellant activity and binds specifically to octopamine receptors^51^. Also, it was suggested that amitraz has agonistic effect against TyrR^52^. Amitraz and tyramine have *K*_*i*_ values equal to 2.53 and 1.4 *μ*M in *Drosophila* TyrR1, respectively^53^. While the calculated *K*_*i*_ values from the docking results of amitraz and tyramine to SoTyrR were 1.59 and 85.4 *μ*M, respectively. On the other hand, the amino acid residues that participate in the molecular recognition of tyramine in TyrR were previously reported based on a mutation study done with *Bombyx mori* TyrR^14^. The docking results here have confirmed the importance of Asp (Residue 114) at TM 3 in forming H-bonding with the amino group of tyramine. However, other interactions with amino acids noted in the study did not appear in the docking results. Qualitatively, it can be suggested that the binding pocket for tyramine ligand has other possible important amino acid residues aside from the conserved motif of molecular recognition for aminergic receptors. The difference in obtained *K*_*i*_ values and experimentally determined binding sites, especially of tyramine, are surmised to be an effect of varying protein conformational state when the ligand binds to the receptor. It is therefore helpful to employ ensemble docking scheme to MD-derived structures of SoTyrR^54^.

Moreover, GPCRs undergo conformational changes after ligand binding which allow the rearrangement of intracellular domains directly involved in cellular signaling^19,20^. Different protein motions can be observed at various timescales. For instance, localized motions such as fluctuations of chemical bonds, angles, and movement of side chains can be discerned within femto- to nanosecond scale. On the other hand, the reorientation of helices, rearrangement of loops and side chains positions can be observed from nano- to millisecond timescale^55,56^.

The importance of IL3 region in the activation of GPCRs have been studied before with *μ*-opioid receptors^57^, muscarinic^58^, and *β*2-adrenergic^59^. These previous structural and conformational dynamics studies of structurally resolved GPCRs were in agreement that the IL3 serves a special role in terms of overall dynamics. The pocket that is comprised of TM3, TM5, TM7, IL2, and IL3 interacts with the G protein. These domains form polar and hydrophobic interactions with the partner G protein^56^. Upon the binding of activator ligands, the IL3 becomes more stable and the residues of GPCR at the intracellular face would need to find its G protein partner where further activation would be triggered. However, there are no experimental literature suggesting the biophysical effects of agonist-binding on SoTyrR. This study reinforces the idea that IL3 plays a crucial role after agonist ligand binding.

The detailed description of the interplay of the movement of important GPCR domains (e.g. IL3) on the activation process is limited^56^. From these domains, IL3 is known to be highly flexible and typically unresolved in most of the available crystal structures. The stabilization of IL3 region is also highly coupled to the GPCR and G protein coupling where previous studies have probed^27,60,61^. This may signify a highly dynamic nature of IL3. This claim was verified in the RMSD of SoTyrR (6.90 ± 0.48 Å) which decreases after ligand binding in SoTyR-tyramine. IL3, comprised of 158 amino acid residues which connects TM5 and TM6 domains, has been experimentally verified to be involved in the activation of *β*1-adrenergic receptors^62^. Moreover, the other significant shifts in RMSD values were observed in the EL1, EL3, and IL4 domains. Note that there is limited motion in the TM helices (RMSD value ≤3Å) as opposed to the evident flexibility of the intracellular and extracellular loop regions where most important dynamics dwell. The local fluctuations (based on B-factor analyses) have become more pronounced also in amitraz-bound SoTyrR, especially at the IL3 region where B-factor ratio have significantly decreased.

The high correlation of IL3 motions to the binding site’s dynamic behavior has lead to the stabilization of SoTyrR and its natural ligand. This has been shown from the correlation analysis of protein dynamics where more positively correlated motions are found in IL3 region. Although the binding sites of the ligands are within the transmembrane bundle, significant motions in the IL3 region impacts SoTyrR overall behavior. Simulation results showed that tyramine, the natural ligand, appears to stabilize the highly flexible IL3 where B-factor have lower values than in its apo state. It was found also from the simulations that TM3 is an important trigger in the conformational rearrangement of IL3. In this regard, the active form of SoTyrR differs significantly by how amitraz activates SoTyrR where the ligand amitraz, an agonist, seems to disrupt the stabilization of IL3. Here, the motion of the SoTyrR-amitraz IL region is highly correlated to the dynamic behavior of TM6 as revealed from the 3D B-factor projection of the complex.

Furthermore, there is a significant difference between the number of states visited by the activation/deactivation processes in SoTyrR-tyramine and SoTyrR-amitraz based on the network analysis. PCA have revealed that it took longer time for the amitraz to reach its structural convergence unlike in tyramine that took only 20 ns. Porcupine plots of PCs 1, 2, and 3 have also shown the connections of agonist-binding to the large displacements of IL3 region. PC 1 correlates to this observed IL3 displacement which results in corresponding shifts in RMSD and B-factor values for SoTyrR-tyramine complex.

Although the timescale of simulations limits the full mechanistic understanding of the whole SoTyrR, the 200-ns MD simulations can already recognize the early effects and small differences between the two agonists’ activation processes with focus on the IL regions. This also gives us an insight on the important conformational changes of the receptor that can push higher propensity of Gprotein binding to its IL region.

## Conclusion

Tyramine receptors are important signaling proteins present in invertebrates. Here, we probed the structural dynamic effects of agonist ligands tyramine and amitraz to SoTyrR. We have provided the most probable binding site for tyramine and amitraz by modeling the 3D structure of the insect receptor and by ensemble docking of the agonists. Based on the molecular docking results, we had verified the interaction of Asp114 in the molecular recognition of tyramine in SoTyrR consistent with experimentally determined binding studies in TyrR. The most common feature of the ligand bindings, especially amitraz, is that they are highly hydrophobic in nature based on the residues comprising the transmembrane helices bundle. Results from independent MD simulations of SoTyrR-ligand complexes revealed the importance of transmembrane’s dynamic behavior to the IL3 domain movements^27,60,61^. The B-factor and RMSD behavior of the SoTyrR IL3 domain have increased significantly in the SoTyrR-amitraz and SoTyrR than in SoTyrR-tyramine. From the correlated motions of SoTyrR-amitraz, the IL3 region was found to be the focal point of SoTyrR’s overall dynamics. Moreover, examination of the network profile of different protein-ligand complexes have shown that there are greater number of states visited in the SoTyrR-amitraz complex that can potentially delay or hinder the activation of tyramine receptor. This needs to be explored further in the future by using other enhanced sampling MD methods. The combination of different computational methods has given us means to propose important dynamic features of ligand-dependent conformational changes in GPCR. By these, potential behavior and structural dynamics consequence of agonist ligand binding to the tyramine receptor were proposed and compared between different protein-ligand complexes.

## Methods

In the absence of OAR crystal structures, homology modeling of *Sitophilus oryzae* tyramine receptor (SoTyrR) was employed. The amino acid sequence was obtained from the UniProt database (ID A0A0S1VX60)^63^. The SoTyrR 3D model was generated using the online server I-TASSER^33^.

The protein-membrane complex of SoTyrR was built through the CHARMM-GUI Membrane Builder module^64^. The lipid system is composed of 130 and 121 1-palmitoyl-2-oleoyl-sn-glycero-3-phosphocholine (POPC) at the upperleaflet and lowerleaflet of the bilayer membrane, respectively, along with 0.15 M KCl salts and 17,582 water molecules.

All-atom molecular dynamics simulations in each protein-ligand complex were performed. Energy minimizations, heating, equilibration, and production runs were accomplished using NAMD 2.11^41^ using AMBER ff14SB^65^, Lipid14^66^, TIP3P^67^, and general AMBER force field (or GAFF)^68^ for the protein, lipid, water, and ligands, respectively. Tyramine and amitraz were parameterized using the Antechamber package^69^ from AMBER 14^70^. Minimization was done for 10,000 steps followed by heating from 0 to 300 K with 10 K increment per step. Particle mesh Ewald with periodic boundary conditions was used for the calculation of electrostatic interactions^71^ and Langevin dynamics was applied for temperature control at 300 K^72^. Dataframes were collected at 2 fs interval for the whole 200 ns production run.

Here, tyramine and amitraz ligands were docked to SoTyrR. AutoDock VINA was used for blind ensemble molecular docking of the target ligands with search box size 40 × 40 × 30 Å^3^ and centered at (−3.242, −2.16, −28.905) covering the extracellular region and upper transmembrane helices^38^. An ensemble of SoTyrR protein structures was obtained from the 100-ns simulation in aqueous lipid bilayer and the binding energy was reported as an average of the ensemble docking results. The starting structures for the MD simulations were based on the lowest binding energy obtained from the ensemble docking.

Analyses of MD generated trajectory files were done using cpptraj^73^. Root-mean-square deviation was determined by using the first frame of SoTyrR-apo production as reference. B-factor or the Debye-Waller factor was extracted from the full production runs and calculated using the equation: $B=[8{\pi }^{2}(\Delta {r}^{2})]/3$ where *r* is the coordinate of the atom^74^. The B-factor ratio of ligand-bound SoTyrR and SoTyrR-apo was also determined and projected in the 3D structures using Chimera^75^.

To assess the reproducibility of simulations results, we ran parallel all-atom MD simulations. A coarse-grained SoTyrR in POPC lipid bilayer was built in CHARMM-GUI Martini Maker server^76^. Coarse-grained MD simulations (CGMD) was performed in GROMACS 5.1 while using standard MARTINI protocol^77,78^. Representative structures from 1 *μ*s CGMD were extracted and back-mapped to all atom representation using insane.py code from http://cgmartini.nl/images/tools/insane^78^. Using these as initial structures, 50-ns all atom MD simulations were employed, following the same procedure done in this study. The average RMSD for SoTyrR, SoTyrR-tyramine, and SoTyrR-amitraz were reported (Table 2).

To characterize the correlated motions of SoTyrR, Mixed PCA was done by combining snapshots from the SoTyrR and SoTyrR-ligand simulations. We obtained the covariance matrix and performed principal component analyses to these snapshots^43,79^. The covariance matrix was obtained from the atomic fluctuations of the C*α*’*s* and diagonalized to yield eigenvectors and eigenvalues. These represent the direction and magnitude of motions, respectively. The two highest eigenvalues were reported as PC 1 and PC 2 and were projected back to Cartesian coordinate. Mixed PCs from SoTyrR-ligand system were projected with respect to the SoTyrR PCs for comparison as previously done^80^. In total, there are 400 000 snapshots of the SoTyrR and SoTyrR-ligand trajectories (200 000 each). After this, final 200 000 snapshots of each system were projected onto PC 1 and PC 2. Porcupine plot analysis was done using VMD^81^ where the first and last frames of the top PCs were compared.

Clustering analyses were also done to cluster similar protein structures and to identify the transition network between them^82^. A structural cluster is a representation of a local minimum in the energy landscape^83^. Conformational cluster transition network (CCTN) was generated based on the transition between clustered structures^84,85^. Here, K-means clustering algorithm in pytraj was used^73^. The most optimal root-mean-square (RMS) distance value equal to 1.15 Å was applied in the calculation. Then, an edge was added between two vertices of clustered structures. Python library graph-tool was used to represent the networks generated from the clustering algorithm (http://graph-tool.skewed.de/)^86^.

## Supplementary information

Supplementary Information

## Data Availability

The data that support the findings of this study are available from the corresponding author upon reasonable request.
