# Supplementary material for: Ligand-Induced Conformational Dynamics of A Tyramine Receptor from Sitophilus oryzae
Source: Sci Rep. 2019 Nov 7;9:16275. doi: 10.1038/s41598-019-52478-x (PMC6838067; doi:10.1038/s41598-019-52478-x)
Supplement: Supplementary file 1 — Supplementary Information [file 41598_2019_52478_MOESM1_ESM.pdf]

Supplementary Information

Ligand-Induced Conformational Dynamics of A Tyramine Receptor from *Sitophilus oryzae*

by Mac Kevin E. Braza, Jerrica Dominique N. Gazmen, Eizadora T. Yu, and Ricky B. Nellas

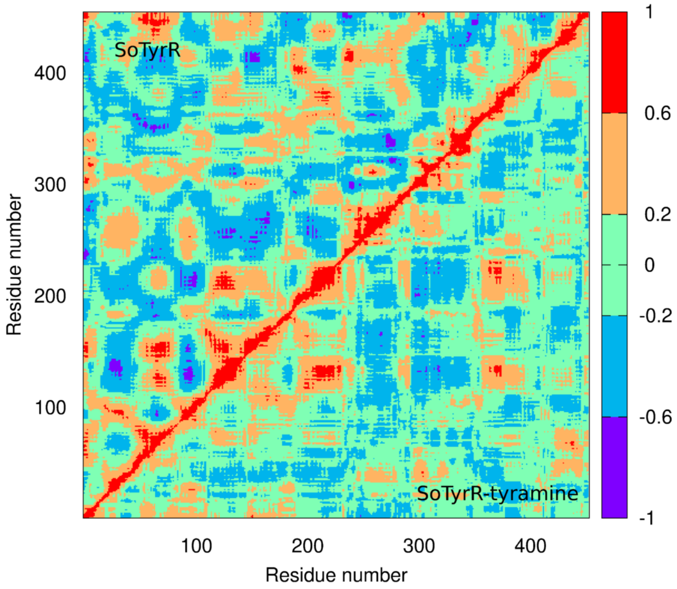

(a)

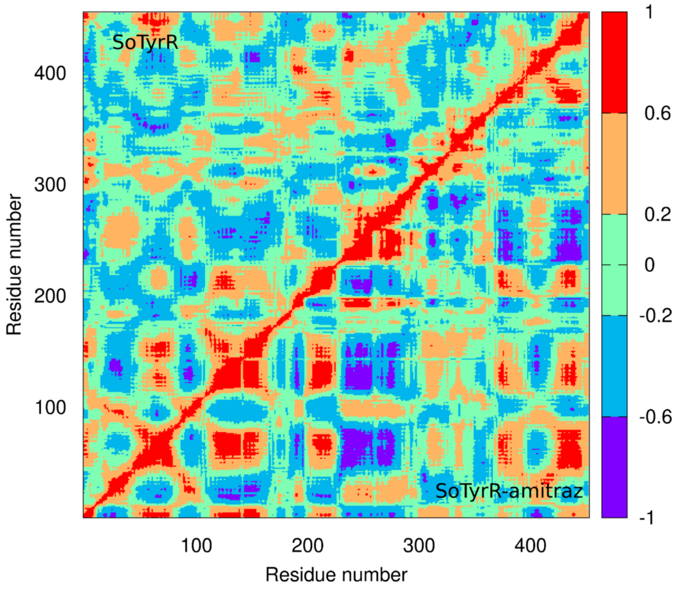

(b)

SI Figure 1

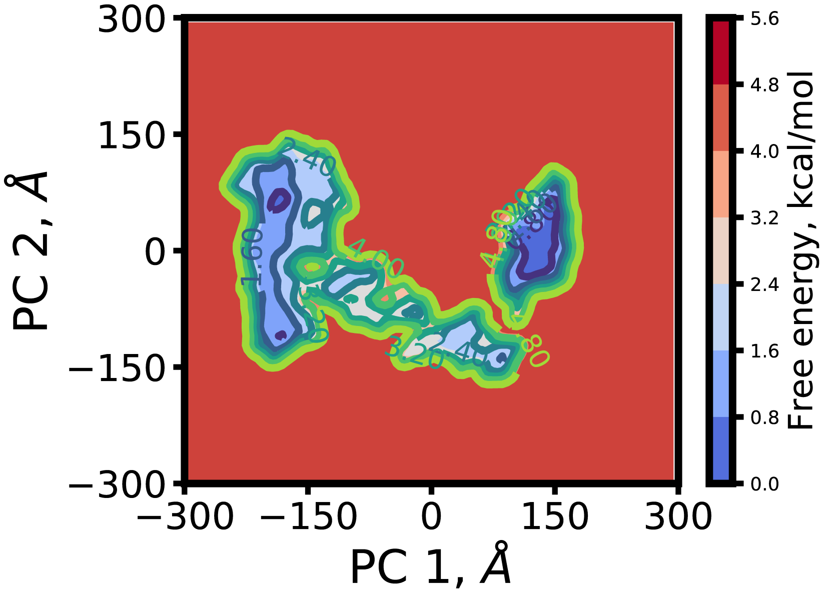

(a)

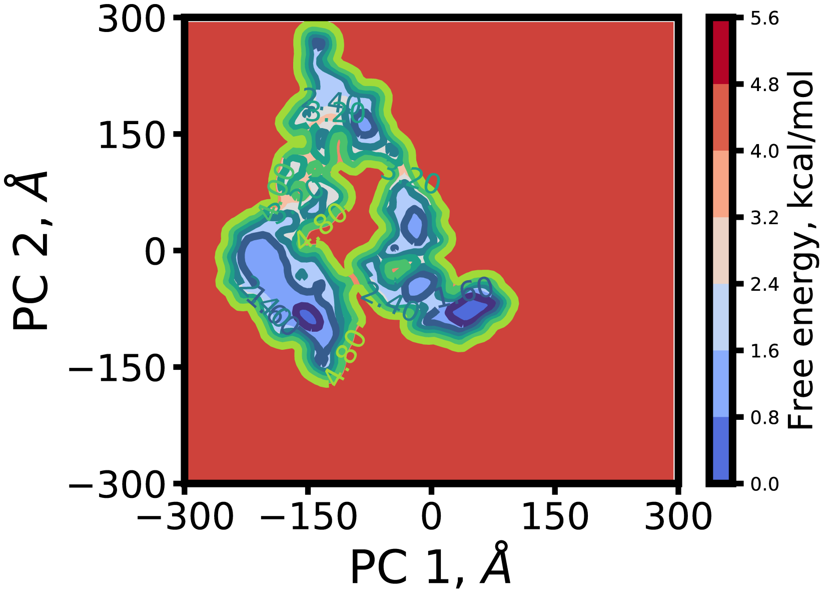

(b)

SI Figure 2

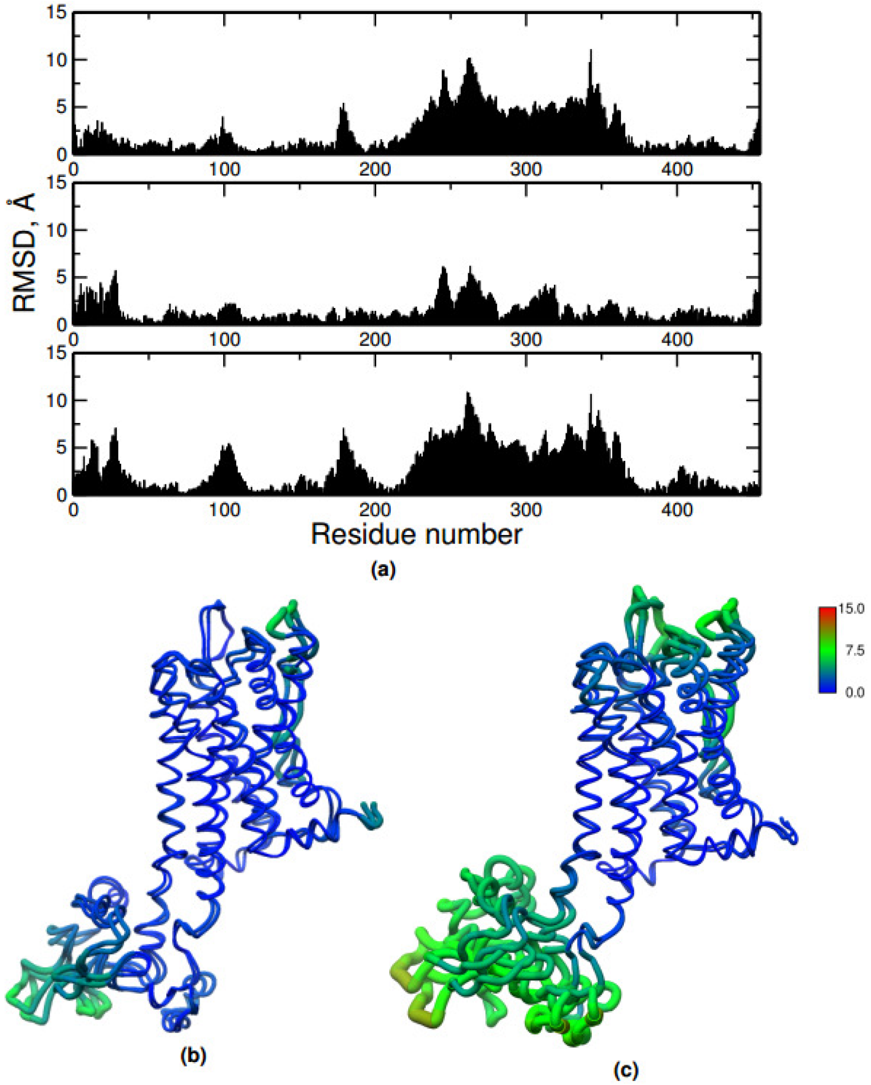

SI Figure 3

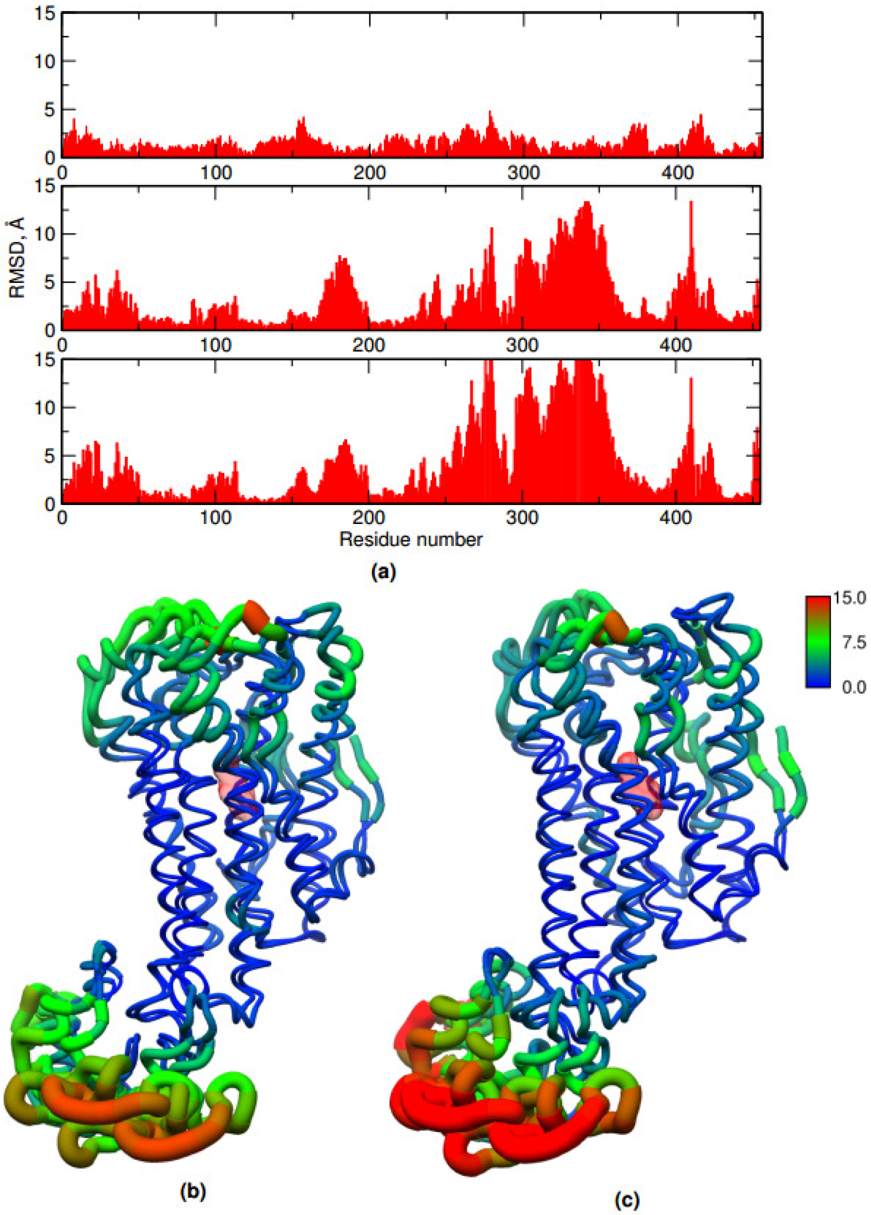

SI Figure 4

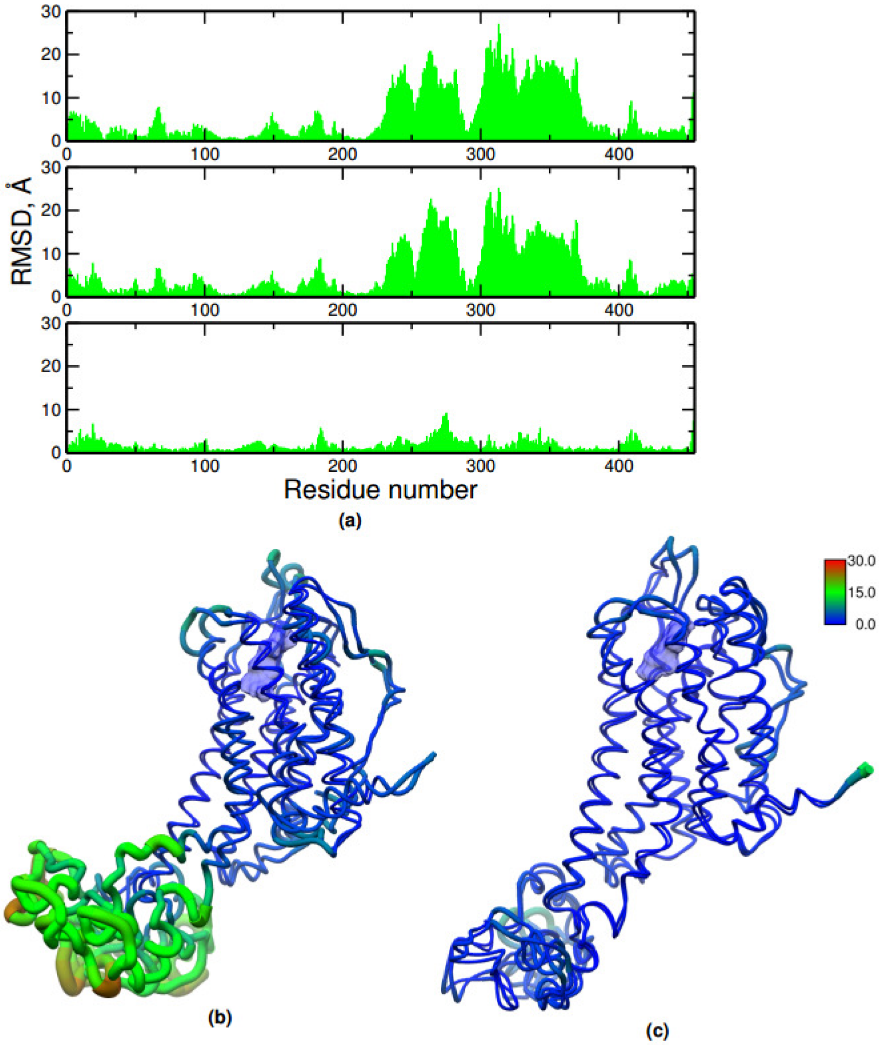

SI Figure 5

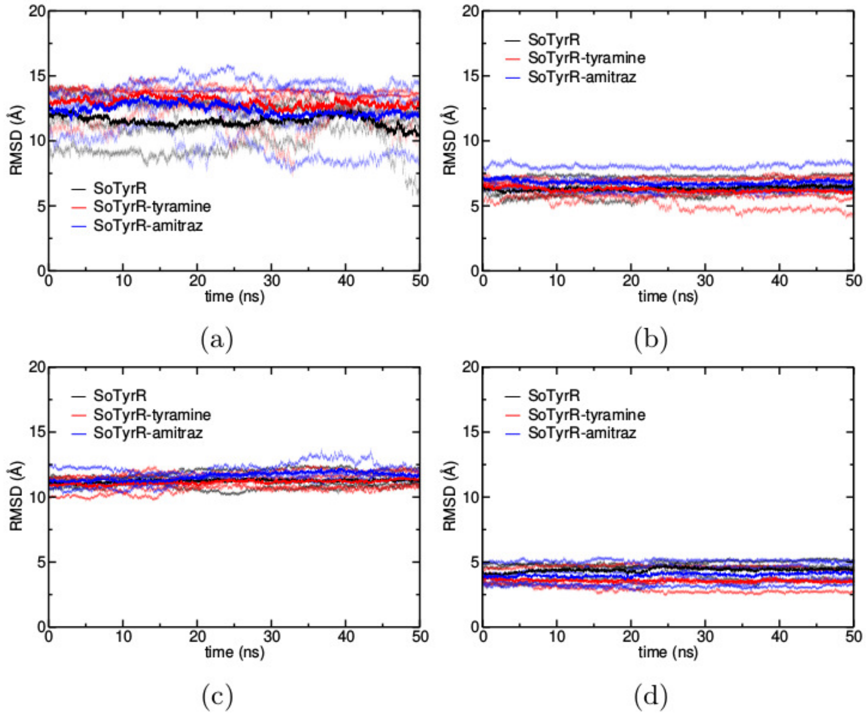

SI Figure 6

List of Supplementary Information Figures

SI Figure 1. Correlation map of SoTyrR and SoTyrR-ligand displayed as triangle forms. The upper half and lower half triangle display the apo and ligand (a) tyramine, and (b) amitraz correlated dynamic behavior of SoTyrR amino acid residues, respectively.

SI Figure 2. Free energy plot of the principal component analyses for (a) SoTyrR-tyramine:SoTyrR and (b) SoTyrR-amitraz:SoTyrR. The two PCs in SoTyrR-tyramine and SoTyrR-amitraz are projected with respect to SoTyrR PCs.

SI Figure 3. (a) RMSD of structures from nodes 1 and 2, 1 and 3, 2 and 3 (from top to bottom) in SoTyrR network analysis. Superimposition of nodes (b) 1 and 3, (c) 2 and 3. The thickness of worm representation depicts the RMSD value between the two structure. The thicker the structure the higher the value of RMSD. RMSD color scale bar is reported in Å.

SI Figure 4. (a) RMSD of structures from nodes 1 and 2, 1 and 3, 2 and 3 (from top to bottom) in SoTyrR-tyramine network analysis. Superimposition of nodes (b) 1 and 3, (c) 2 and 3. The thickness of worm representation depicts the RMSD value between the two structure. The thicker the structure the higher the value of RMSD. RMSD color scale bar is reported in Å.

SI Figure 5. (a) RMSD of structures from nodes 1 and 2, 1 and 3, 2 and 3 (from top to bottom) in SoTyrR-amitraz network analysis. Superimposition of nodes (b) 1 and 3, (c) 2 and 3. The thickness of worm representation depicts the RMSD value between the two structure. The thicker the structure the higher the value of RMSD. RMSD color scale bar is reported in Å.

SI Figure 6. Average RMSD of SoTyrR extracellular loops (EL) and intracellular loops domains in 50-ns all atom MD simulations. SoTyrR (black), SoTyrR-tyramine (red), and SoTyrR-amitraz. (a) EL1; (b) EL2; (c) IL3; and (d) IL4.
